# Carotenoid Content in Breastmilk in the 3rd and 6th Month of Lactation and Its Associations with Maternal Dietary Intake and Anthropometric Characteristics

**DOI:** 10.3390/nu11010193

**Published:** 2019-01-18

**Authors:** Monika A. Zielinska, Jadwiga Hamulka, Aleksandra Wesolowska

**Affiliations:** 1 Department of Human Nutrition, Faculty of Human Nutrition and Consumer Sciences, Warsaw University of Life Sciences—SGGW, 159 Nowoursynowska St., 02-776 Warsaw, Poland; monika_zielinska@sggw.pl; 2 Laboratory of Human Milk and Lactation Research at Regional Human Milk Bank in Holy Family Hospital, Department of Neonatology, Faculty of Health Sciences, Medical University of Warsaw, 63A Zwirki i Wigury St., 02-091 Warsaw, Poland; aleksandra.wesolowska@wum.edu.pl

**Keywords:** bioactive factors, carotenoids, dietary intake, high-performance liquid chromatography (HPLC), human milk, lactation, maternal diet, prospective study

## Abstract

Carotenoids are diet-dependent milk components that are important for the visual and cognitive development of an infant. This study determined β-carotene, lycopene and lutein + zeaxanthin in breastmilk and its associations with dietary intake from healthy Polish mothers in the first six months of lactation. Concentrations of carotenoids in breastmilk were measured by HPLC (high-performance liquid chromatography) (first, third, sixth month of lactation) and dietary intake was assessed based on a three-day dietary record (third and sixth month of lactation). The average age of participants (*n* = 53) was 31.4 ± 3.8 years. The breastmilk concentrations of carotenoids were not changed over the progress of lactation. Lycopene was a carotenoid with the highest content in breastmilk (first month 112.2 (95% CI 106.1–118.3)—sixth month 110.1 (103.9–116.3) nmol/L) and maternal diet (third month 7897.3 (5465.2–10329.5) and sixth month 7255.8 (5037.5–9474.1) µg/day). There was a positive correlation between carotenoids in breastmilk and dietary intake (lycopene *r* = 0.374, *r* = 0.338; lutein + zeaxanthin *r* = 0.711, *r* = 0.726, 3rd and 6th month, respectively) and an inverse correlation with maternal BMI in the third month of lactation (β-carotene: *r* = −0.248, lycopene: *r* = −0.286, lutein + zeaxanthin: *r* = −0.355). Adjusted multivariate regression models confirmed an association between lutein + zeaxanthin intake and its concentration in breastmilk (third month: *β* = 0.730 (0.516–0.943); 6th: *β* = 0.644 (0.448–0.840)). Due to the positive associations between dietary intake and breastmilk concentrations, breastfeeding mothers should have a diet that is abundant in carotenoids.

## 1. Introduction

Breastfeeding is the best feeding method for newborns, infants and toddlers. International organizations (e.g., WHO, UNICEF, AAP, ESPGHN) recommend exclusive breastfeeding during the first six months of life and further breastfeeding along with complementary feeding up to two years or more, as long as the infant and mother desire [1,2,3]. Exclusive breastfeeding at this time provides sufficient energy and macronutrients as well as most of the micronutrients (except vitamin D and K) to meet the mean requirements of healthy term-born infants [3,4]. During this time, breastmilk provides not only necessary nutrients, however also a variety of bioactive factors, such as immunoglobins, stem cells, cytokines, hormones, growth factors and phytochemicals (e.g., carotenoids and flavonoids) [5,6,7]. These compounds are crucial for optimal development and further health, including decreased risk of infectious diseases during childhood as well as chronic non-communicable diseases throughout the lifespan [2,8]. Previous studies have shown that breastmilk composition is influenced by many determinants, including maternal factors (e.g., age, nutritional status, dietary intake, tobacco use or passive smoking), infant factors (gestational age, age, gender) and physiological factors (lactation stage, nursing stage, diurnal variation) [9,10,11,12,13,14]. The content of some nutrients in breast milk is dependent on maternal dietary intake, including fatty acid profile [15], iodine [16] and vitamins B_1_, B_2_, B_6_ and B_12_ [17]. However, maternal dietary intake has a limited influence on breastmilk macronutrient composition [14] and some micronutrients, e.g., folic acid [17] and zinc [18]. Some studies have indicated that maternal nutritional status measured as body mass index (BMI) may influence the milk fat concentrations [14] as well as carotenoid concentrations [19].

Carotenoids (β-carotene, lycopene, lutein, zeaxanthin, β-cryptoxanthin, astaxanthin) are plant bioactive compounds that cannot be synthesized by mammals however are supplied with a high dietary intake of vegetables and fruits (especially green, yellow, orange or red) or some animal products (e.g., yolk, salmon or rainbow trout) [20,21,22]. Numerous studies in recent years have shown their positive impact on human health, mainly in adults [23,24,25], however also during pregnancy and early childhood, as noted previously [6,26]. Recent studies focusing on breastmilk carotenoids have shown that their concentration depends on maternal dietary intake [27] and varies among populations [19,28,29,30], especially due to different dietary habits. Other factors may also affect the carotenoid concentration in breast milk, such as education status or anthropometric parameters of mothers [19]. Giordano and Quadro [26], in a recent review, emphasize the importance of monitoring maternal and infant lutein and zeaxanthin status due to its importance during pregnancy and the neonatal period [26]. An analysis of breastmilk carotenoids may be a useful, non-invasive method due to the correlations between maternal carotenoid status, breastmilk carotenoids and infant carotenoid status [27,30,31,32,33]. To date, few studies have considered the impact of dietary intake of carotenoids and maternal factors on breastmilk carotenoid concentration and there is a lack of long-term studies on the subject. 

The aims of this study were (1) to investigate the concentrations of selected carotenoids (β-carotene, lycopene and lutein + zeaxanthin) in breastmilk from healthy mothers living in an urban area of Poland in the first six months of lactation (2) to determine the dietary intake of carotenoids in the third and sixth months of lactation and (3) to explore the associations between breastmilk carotenoids and dietary intake and other maternal factors.

## 2. Materials and Methods

### 2.1. Ethical Approval

The study was approved by the Ethics Committee of the Medical University of Warsaw in 2015, Resolution No. AKBE/139/15. Written consent was obtained from all participants and the study was conducted in compliance with the Helsinki Declaration.

### 2.2. Study Design

The study consisted of three study sessions at the first, third and sixth month of lactation. All mothers completed a questionnaire which included detailed questions about lifestyle, sociodemographic, health factors and nutrition (e.g., maternal education, household income, dietary supplement use) during the pre-conceptional, prenatal and postnatal periods. At each study visit, breastmilk samples were also collected and anthropometric measurements were assessed in infants and mothers, maternal psychological status was evaluated, as well as the infant psychomotor development in the sixth month. Detailed information about the study design is shown in Table 1. The data used in this paper are part of the data that were obtained during this study.

### 2.3. Study Participants

**Figure 1:**
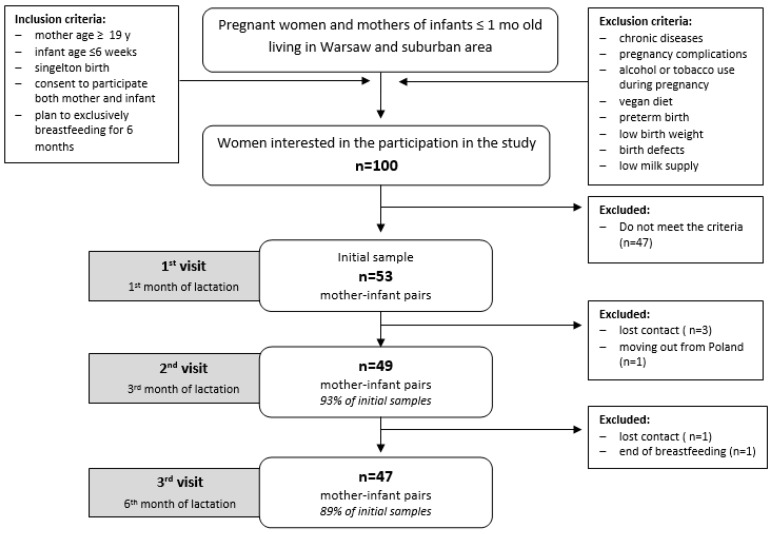
Flowchart: study design and study sample collection.

Recruitment was conducted between April 2015 and July 2017 in the Holy Family Hospital in Warsaw among patients of Obstetrics Clinic as well as breastfeeding women from the local community using social media groups. Eligibility criteria included women between 18 and 45 years of age giving birth to a single, healthy infant. The exclusion criteria included maternal chronic disease (kidney, liver, gastrointestinal diseases influencing nutrient absorption, hypertension, diabetes type I or II) and pregnancy complications (including preeclampsia or eclampsia, pregnancy induced hypertension). The study also included 53 mother-infant pairs (Figure 1). The mean age of mothers (*n* = 53) was 31.4 ± 3.8 years. Most of the mothers had a university education and a high average income. Before pregnancy, most of them had a normal BMI (*n* = 46; 87%); at the first month of lactation, 78% were classified as having normal body weight (*n* = 40), 86% (*n* = 42) at the third month and 85% (*n* = 40) at the sixth month. Further, 25 participants (47%) were primiparas and 53% of the infants were females (*n* = 28; detailed study group characteristics are shown in Table S1). Most of the study visits took place during the spring-summer period (no statistically significant differences between the number of visits in particular seasons, *p* = 0.482).

### 2.4. Breastmilk Collection

Breastmilk samples were collected by participants at home after being given detailed instructions. The same amount (5–10 mL) of pre-feeding and post-feeding breastmilk was collected 24 hours prior to each study visit at four time periods (06:00–12:00; 12:00–18:00; 18:00–24:00; 24:00–06:00) to minimize the daily differences in milk composition due to the 24-hour variation in carotenoid concentrations that is described in literature [39,40]. The samples were obtained by a breast pump or manually from the breast(s) that the infant fed from. Breastmilk samples were transported to the Holy Family Hospital in Warsaw under cooling conditions. Next, the same amount from all four samples was collected and mixed in a Vortex shaker IKA MS2 (IKA Works Inc., Wilmington, North Carolina, USA) for one minute. The pooled sample was distributed in 2 mL clear polypropylene tubes, labelled and stored at −80 °C for later analysis within six months of collection. Precautions were taken throughout the breastmilk sample collection and procedures to minimize the exposure of samples to temperature, light and air.

### 2.5. Breastmilk Composition Analysis

Breastmilk energy value and fat content per 100 mL of the raw sample of breastmilk were analysed using a MIRIS human milk analyser HMA (Miris, Uppsala, Sweden). Prior to the analysis, each sample (*n* = 149) was warmed to 40 °C and homogenized (1.5 s/1 mL of sample) using a sonicator (milk homogenizer, Miris, Uppsala, Sweden) according to the method described previously [14]. From each raw pooled sample, macronutrient composition was analysed three times (~2 mL per analysis) and the average of the three measurements was used for further statistical analysis.

### 2.6. Breastmilk Carotenoid Analysis

Carotenoid concentration (β-carotene, lycopene and lutein + zeaxanthin) in milk samples was assessed using high-performance liquid chromatography (HPLC). Milk samples for analysis were prepared based on the modified method published by Macias and Schweigert [41]. To 2 mL of milk, 500 μL of a 12% solution of pyrogallol, 50 μL, 1% ascorbic acid in 0.1 M HCl, 1.5 mL 30% KOH solution and 2.5 mL ethanol were successively added. The mixture was shaken for 30 seconds with a Vortex shaker and was then incubated in a 50 °C water bath for 40 minutes. Subsequently, 1 mL of saturated NaCl solution and 1 mL of *n*-hexane were added to the ice-cooled samples, followed by vigorous shaking for three minutes. The sample was then centrifuged for 10 minutes at 4 °C (8000 rpm). Immediately after centrifugation, the upper hexane layer was transferred to a new tube. The extraction was repeated two more times. The combined hexane extracts were evaporated in a vacuum evaporator (30 minutes at 30 °C). The formed precipitate was dissolved in 0.5 mL of hexane and was transferred to dark glass vials. 

The content of individual carotenoids in the prepared milk samples was determined using the Shimadzu HPLC system (Japan: 2 LC-20AD pumps, CMB-20A controller system, SIL-20AC autosampler, UV/IS SPD-20AV detector, CTD-20AC controller) using C18 Synergi Fusion-RP 80i columns (250 × 4.60 mm, Phenomenex, CA, USA). The determination of the studied carotenoids was carried out at a wavelength of 471 nm for lycopene, 450 nm for β-carotene and 445 nm for lutein + zeaxanthin. The mobile phase consisted of two phases—phase A: acetonitrile/methanol mixed in proportions of 90:10 (90/10; *v*/*v*) and phase B: methanol/ethyl acetate, in a 34:16 ratio (34/16; *v*/*v*). The flow rate of the developing mixture was 1.0 cm^3^/min and the sample injection was 100 μL. Individual carotenoid concentrations in milk were calculated by comparing them with a corresponding standards curve (standards of catalogue numbers: β-carotene C4582, lutein + zeaxanthin X6250, lycopene L9879 from Sigma-Aldrich Inc., Merck KGaA, Darmstadt, Germany). The standard curves for all carotenoids are shown in supplementary materials (Figure S1). The concentrations of the studied carotenoids were expressed in nmol/L. Since lutein and zeaxanthin could not be completely resolved and were summed, all references to milk lutein concentrations refer to lutein + zeaxanthin.

### 2.7. Dietary Intake Analysis

The maternal diet at the 3rd and 6th month of lactation was assessed using the 3-day dietary record conducted by respondents before the study visits (on three typical days, two weekdays and one weekend day). Respondents received all necessary information and instructions on conducting a dietary record, including the importance of scrupulosity recording all consumed foods and beverages during the period covered by the 3-day dietary records. During the recording, respondents used a kitchen scale to measure the weight of the serving, or if they did not have kitchen scales, they estimated the servings using typical household measures. All sizes of portions were verified using the “Album of Photographs of Food Products and Dishes” created by the National Food and Nutrition Institute [42]. All nutritional data were collected and analysed by the qualified dietician.

Based on the data from the 3-day record of daily energy value and dietary intake of selected macronutrients (protein, carbohydrates, fat) and micronutrients (vitamin A, E, D, B_1_, B_2_, B_3_, B_6_, B_12_, C, folic acid; iodine, calcium, potassium, phosphor, magnesium, iron, zinc, copper, manganese), the dietary fibre and fatty acid profile were calculated using Dieta 5.0 Software (National Food and Nutrition Institute, Warsaw, Poland) based on Polish food-composition tables [43]. The total dietary intake of selected carotenoids (lutein + zeaxanthin, lycopene and β-carotene) were estimated using data from the USDA Database [22]. In addition, data on dietary supplement use in the preconceptional prenatal and postnatal periods were collected including the used dose, name and the brand of dietary supplement. The data were used to calculate the average daily nutrient intake with dietary supplements. The intake of nutrients was calculated as a mean from the three recorded days to obtain the mean daily intake from diet, dietary supplements and the sum of both for each participant. For the purpose of this study, we used only energy value, fat intake and intake of antioxidant fat-soluble vitamins (A and E) and carotenoids (lutein + zeaxanthin, lycopene and β-carotene) for analysis.

### 2.8. Anthropometric Measurements

The body weight (kg) and height (cm) of mothers were measured according to the International Standards for Anthropometric Assessment [44] to the nearest 0.1 kg or 0.1 cm, respectively, using a professional stadiometer (Seca 799, Hamburg, Germany). All measurements were taken in light clothing and without shoes. Body mass index (BMI, kg/m^2^) was calculated and classified according to the World Health Organization (WHO) [45]. BMI and body mass changes between visits were calculated. 

### 2.9. Statistical Analysis

The parameters that were analysed in this study were presented as: means and standard deviation or 95% coefficient intervals (CI), minimum and maximum values. To assess the normality of distributions, the Shapiro–Wilk test was applied. The differences between groups were analysed using the Wilcoxon matched pairs test (for two repeated measurements) or the Friedman ANOVA test (for three repeated measurements). For qualitative variables, the Cochrane Q test was used to check the differences between three repeated measurements and the Chi^2^ McNemar test was used for the two repeated measurements. 

The correlations between breastmilk carotenoids and the maternal anthropometric and sociodemographic characteristics, as well as dietary intake, were estimated with Spearman’s rank or Kendall’s *tau* correlations (if one of the variables was measured on an ordinal scale) coefficient. Linear regression was used to investigate the relationship between breastmilk carotenoids and the dietary intake of its carotenoids. The one univariate and final three multivariate models were specifically adjusted for season, maternal age, BMI, education, mode of delivery, fat, vitamin E and vitamin A intake. All of the analyses were performed using Statistica 13.1 software (Dell Inc., Round Rock, TX, USA). For all of the tests, *p* ≤ 0.05 was considered as significant. 

## 3. Results

### 3.1. Breastmilk Composition and Carotenoid Concentration

The average energy value, fat content and carotenoid concentrations in breastmilk at the 1st, 3rd and 6th month of lactation are shown in Table 2. The energy value and fat content in breastmilk were, on average, around 68–69 kcal/100mL and 3.8–3.9 g/100mL, respectively, and this level was similar regardless of the lactation month. The highest concentration of determined carotenoids was observed for lycopene (minimum–maximum values 77.2–176.9 nmol/L). The concentrations of β-carotene and lutein + zeaxanthin were at similar levels—mean values around 33 nmol/L (despite the insignificant 12% higher concentration of lutein + zeaxanthin at the sixth month due to the higher dietary intake at this month) and also did not differ between the months of lactation.

### 3.2. Dietary Intake

Table 3 shows the energy value and dietary intake of selected nutrients at the third and sixth month of lactation. There was no statistical difference between the third and sixth month for energy value and fat intake. We observed statistically significant differences between total vitamin E intake (dietary supplements and diet; *p* = 0.000) and for vitamin A, both dietary and dietary with supplements (*p* = 0.010 and *p* = 0.009, respectively). These differences are associated with the higher use of dietary supplements in the third month of lactation compared with the sixth month (90% vs. 81%; *p* < 0.001). The highest consumption was recorded for lycopene, followed by β-carotene and lutein + zeaxanthin in the lowest amount. We observed no significant differences in carotenoid intake between the months of lactation.

### 3.3. Associations between Carotenoid Concentrations, Dietary Intake and Maternal Characteristics

The associations between carotenoid concentrations, maternal factors and dietary intake are shown in Table 4. The intake of all of the analysed carotenoids positively correlated with its carotenoid breastmilk concentrations at the third and sixth months. For lutein, we observed a strong correlation (third month: *r* = 0.711, sixth month: *r* = 0.726; *p* ≤ 0.001), for β-carotene—a weak and moderate correlation (third month: *r* = 0.442, sixth month: *r* = 0.532; *p* ≤ 0.001), whereas for the lycopene, only a weak correlation (third month: *r* = 0.374; *p* ≤ 0.01, sixth month: *r* = 0.338; *p* ≤ 0.05). We found statistically significant adverse correlations between maternal BMI (kg/m^2^) and all three carotenoid concentrations in breastmilk at the third month of lactation (β-carotene *r* = −0.337; lycopene *r* = −0.286; lutein + zeaxanthin *r* = −0.355; *p* ≤ 0.05) and also β-carotene at the sixth month (*r* = −0.337; *p* ≤ 0.05). However, the analysis based on BMI categories found significant inverse associations only for β-carotene at the sixth month (*r* = −0.453; *p* ≤ 0.05). 

Table 5 presents the results of a linear regression analysis of carotenoid concentrations in breastmilk and its dietary intake. The univariate analysis reveals significant associations between dietary individual carotenoids intake in all analysed compounds. After the adjustments (models 2–4), we also found significant associations between dietary intake and breastmilk lycopene and β-carotene at the third month of lactation, however the models were not significant and explained only 3–14% of the variance in breastmilk carotenoids. However, even after adjustment for maternal age, BMI, education and mode of delivery, fat and vitamin A and E intake (model 4), dietary intake of lutein + zeaxanthin explained 51–68% of the variation at both times (third month, *β* = 0.730 (95% CI 0.516–0.943), *p* = 0.000; sixth month *β* = 0.644 (95% CI 0.448–0.840)) and β-carotene explained 35% of the variation in breastmilk β-carotene at the sixth month (*β* = 0.428, 95% CI 0.180–0.676).

## 4. Discussion

In this study, we found that the breastmilk carotenoid concentrations were unchanged through the first six months of lactation. The carotenoid with the highest concentration in breastmilk was lycopene, whereas the β-carotene and lutein + zeaxanthin contents were similar. In addition, lycopene was the main dietary carotenoid. We observed a strong positive relationship between lutein + zeaxanthin dietary intake and its concentration in breastmilk, even after adjustment for confounders. In addition, we noted adverse associations between the breastmilk carotenoids in the third month of lactation and maternal BMI. 

Previous studies investigated the breastmilk carotenoid changes during lactation and found that their concentrations decreased with the duration of lactation [19,31,41,46]. Colostrum contains significantly higher concentrations of carotenoids compared to mature milk, regardless of dietary intake or plasma carotenoid concentration [19,30,31,41,46,47]. However, in our study, we did not find any differences in carotenoid concentration between the first and sixth month of lactation which may be explained by the fact that we analysed only mature milk. According to other studies, the decrease occurs only between colostrum and mature milk and the mature milk then has a stable level of carotenoids [30,31,47]. This difference in carotenoid concentration between colostrum and mature milk suggests the occurrence of a special mechanism of transporting carotenoids into the mammary gland during the first day postpartum [30,41,46,48]. This may be associated with the importance of carotenoids, especially lutein, to the health and development of newborns from the first days of life, including decreasing oxidative stress, as well as protection of the retina and participation in its proper development [6,24,26]. Other studies reported not only longitudinal changes in carotenoid concentration, however also diurnal changes, and even within a feeding session, there were changes as a result of changes in breastmilk fat concentrations [39,40,49].

Previous research has demonstrated that the primary carotenoids in breastmilk are β-carotene, lutein and zeaxanthin, lycopene, α-carotene and β-cryptoxanthin, although differences in their proportions were observed [19,28,29,30,31,39,41,46,50]. In the current study, lycopene was the main breastmilk carotenoid with a concentration of 112.2 at the first month and 110.1 nmol/L at the sixth month. However, studies from other populations reported much lower concentrations of lycopene in mature milk, from 14.0 to 59.8 nmol/L (German [31,41], Brazil [50], China [19,28,30] Australia [28], Canada [28], Chile [28], Japan [28], Mexico [28,30], Philippines [28], UK [28], USA [28,30]). In addition, some studies found much higher concentrations of lutein compared to our results (33.0 and 37.1 nmol/L in the first and sixth month of lactation, respectively), from 44.0–114.4 nmol/L (studies conducted in Chile [28], China [28,30,33], Japan [28], Mexico [28,30]), and some reported lower 6.0–29.0 nmol/L (studies conducted in Germany [41], Brazil [50,51], Australia [28], Canada [28], USA [28], UK [28], China [19]). Similarly, other authors observed higher concentrations of β-carotene 36.2–78.2 nmol/L (Germany [31,41], Australia [28], Canada [28], Chile [28], China [28,30], Japan [28], Mexico [28], UK [28], USA [28,30]), although some studies showed a lower concentration of 16.0–22.0 nmol/L (Philippines [28], Brazil [50,51], China [19]). These differences can be explained by variations in dietary habits between populations, including the availability of fruit and vegetables, as well as preferred dish preparation methods. It is also important to note that other studies use different methods of breastmilk sample collection (for example, total volume of one breast or just 5–12 millilitres of foremilk which had 25% lower concentrations of carotenoids compared to the hindmilk [40]). Breastmilk carotenoids are the only source of carotenoids for newborns and infants because infant formula are not fortified with them [6]. β-carotene has pro-vitamin A properties, so it can be converted, if necessary, to contribute to meeting the nutritional requirement for vitamin A [52]. A growing body of studies highlight the importance of lutein during the neonatal and infancy period due to its role during neuro and visual development, as well as reducing oxidative stress and the risk of disorders associated with prematurity [6,7,24,26]. However, lycopene may also decrease the risk of adverse neonatal outcomes (birth parameters, Respiratory Distress Syndrome, Newborn Intensive Care Unit admission), as shown in a recently published study which assessed the maternal serum lycopene during pregnancy in 180 maternal-infant pairs from the USA [53].

As carotenoids cannot be synthesized by mammals, all breastmilk carotenoids are diet-derived. The source of carotenoids are vegetables and fruits, especially tomatoes and its products for lycopene, green, yellow and orange for lutein and yellow and orange for β-carotene [24]. In our study, lycopene was not only the main carotenoid in breastmilk, however it was also the carotenoid with the highest intake in our study group (7897.3, 7255.8 µg/d, third and sixth month of lactation, respectively), followed by β-carotene (4480.0, 3441.9 µg/day) and lutein + zeaxanthin (2945.2, 3739.3 µg/day). These amounts were similar to the carotenoid intake during the first and second trimester that was reported in a study that was conducted in the USA [54]. β-carotene intake was also similar to the results of a study that was conducted among pregnant Polish women, however the authors reported a lower intake of lycopene and lutein + zeaxanthin [55], although other studies of pregnant women reported a much lower intake of these carotenoids [56,57,58,59]. The NHANES study also showed a much lower lutein intake by women who were at reproductive age [60], although a recent study that was conducted in Canada found a similar intake of lycopene and lutein + zeaxanthin and higher intake of β-carotene [61]. Detailed data on the dietary intake of carotenoids during lactation are limited. Cena et al. [27] calculated lutein consumption in 15 Italian women at the third and 30th day postpartum, which was 1209 ± 157 and 1258 ± 102 µg/day, which is also lower compared to our study. However, this data was obtained almost 10 years ago and dietary habits may have changed since then due to the increasing availability of fruits and vegetables. A study that was conducted among Chinese populations found an intake of lutein + zeaxanthin at the level 3.3 ± 0.41 mg/day [33], which was similar to our study. 

Several studies have found that a dietary intake of carotenoids determines the serum carotenoids [24,27,62] and, hence, it determines the breastmilk concentrations [27,30,31,32,33,50,51]. Other studies analysed serum carotenoids and found strong correlations between maternal plasma and breastmilk, where breastmilk concentrations were 10–120 times lower compared to the serum concentrations [30,51]. Cena et al. [27] described the strong associations between the dietary intake of lutein and its concentration in serum (*r* = 0.94) and breastmilk (*r* = 0.84), however a more recent study by Xu et al. [33] did not find any associations between maternal dietary lutein + zeaxanthin and serum or breastmilk concentrations. Furthermore, a Brazilian study found no association between pro-vitamin A carotenoids and breastmilk β-carotene [51]. Despite the inconsistency of these results, several interventional studies clearly indicated that the concentration of breastmilk carotenoids increases after the consumption of high-carotenoid food products (e.g., carrot or tomato paste, Chlorella) or dietary supplements [62,63,64,65]. A multinational study by Canfield et al. [28] also found that the major milk carotenoids are consistent with major dietary carotenoids. The current study confirmed the associations between dietary carotenoid intake and the breastmilk concentrations that were observed by other authors. Nonetheless, the strength of the association noted in the current study differed depending on the carotenoid—it was weaker for lycopene (*r* = 0.374, *r* = 0.338, third and sixth month, respectively) and β-carotene at the third month of lactation (*r* = 0.442), moderate at the sixth month (*r* = 0.532) and was the strongest for lutein (*r* = 0.711, *r* = 0.726). After adjustment for potential confounders, we confirmed this observation only for lutein and β-carotene. We hypothesized that stronger associations of lutein compared to β-carotene or lycopene were the result of better nutritional economy of lutein and more efficient uptake to the mammary gland and breastmilk due to its important role during early postnatal development [6,26]. Lutein and zeaxanthin, as xanthophylls, are also more polar compared to carotenes and in vitro studies suggest that xanthophylls may have higher bioavailability [30,48]. Due to their end hydroxyl groups, they have a higher polarity and are easily transferred into lipoproteins, which are responsible for carotenoid transport, and further into milk fat globules [30,48]. Milk fat globules are covered by a membrane trilayer that is derived from the endoplasmic reticulum and apical plasma membrane of mammary epithelial cells [30,48,66]. Lutein and zeaxanthin have a higher membrane solubility and a perpendicular orientation within the membrane bilayer which allows them to be more stably bound within the membrane layer compared to carotenes [67]. A recent study comparing dietary and serum carotenoids in men and non-lactating women showed that women have significantly higher concentrations of serum carotenoids than men despite a lower dietary intake of carotenoids, which indicates a sex-difference in the nutritional economy of carotenoids [61]. 

Maternal dietary intake is not the only factor influencing the breastmilk carotenoid status. In the current study, we found adverse associations between all breastmilk carotenoids at the third month of lactation and β-carotene in the sixth month and maternal BMI. A recent study by Xue et al. [19] found that overweight women had lower concentrations of β-carotene compared to women who were of normal weight, however they did not find any associations for lutein, similar to Meneses and Trugo [51] who also did not observe any associations between breastmilk carotenoids and maternal BMI. However, studies analysing the impact of body mass on serum carotenoids found adverse associations between them [61,68]. The explanation of this association could be twofold. Firstly, both being overweight and obese are associated with greater body fat storage and the carotenoids may be uptaken and stored in adipose tissue [48]. A previous study by Johnson et al. [69] showed that women have a higher lutein concentration in adipose tissue compared to men despite a lower dietary intake and similar serum concentration, which also indicates more effective carotenoid accumulation in adipose tissue in women. Previous studies found that circulating concentrations of other micronutrients, e.g., folate and vitamin B_12_, may also be altered in women who are overweight and obese, independent of other maternal factors [70,71]. This observation indicated the possibility of a modification in micronutrient metabolism, including carotenoids, which resulted in reduced plasma levels and increased uptake in other tissues, especially adipose tissue [70,71]. Secondly, being overweight and obese coincide with elevated oxidative stress levels which reduces the carotenoid level, one of the antioxidant compounds [72]. On the other hand, we observed adverse associations only between the BMI category and β-carotene at the sixth month of lactation. However, more research is needed to explain this association during the lactation period as well as the impact of pre-pregnancy and pregnancy diet on the breastmilk carotenoids due to physiological weight loss during the lactation period.

### Strengths and Limitations

The strength of this study is that we used twice repeated three-day records to collect the data on maternal nutrition which helped to decrease the risk of underestimating or overestimating long-term dietary habits. Second, we collected breastmilk samples from a 24-hour period, as well as both foremilk and hindmilk which minimized possible errors due to changes in breastmilk composition. Third, we had a low drop-out rate (11%), despite a relatively long follow-up period. 

Finally, a number of potential limitations need to be considered. First, we had a sample with a very good socio-economic status and education level that were higher than the national average. Our group also had a moderate number of participants which was generally larger than some studies, mainly prospective (*n* = 21 [27,31], *n* = 23 [40], *n* = 46 [51]) and lower compared to other mainly cross-sectional studies (*n* = 56 [33]; *n* = 140 [46]; *n* = 365 [30]; *n* = 456 [28]; *n* = 509 [19]). Second, we used convenience sampling. Due to this factor, precautions must be taken when extrapolating the results. Third, we used three-day records only at the second and third visit, so we cannot analyse the associations between dietary intake and breastmilk carotenoids at the first month of lactation. Fourth, we did not collect maternal serum, so we cannot analyse the relationship between the maternal dietary intake of carotenoids and plasma correlations.

## 5. Conclusions

The concentrations of β-carotene, lycopene and lutein + zeaxanthin in breastmilk samples, as well as its dietary intake, were studied in the first six months of lactation in healthy women from an urban area of Poland. Overall, our findings reveal that maternal dietary intake of carotenoids positively correlates with breastmilk concentrations, especially for lutein. Furthermore, breastmilk carotenoids in the studied population were moderate (β-carotene and lutein + zeaxanthin) or relatively high (lycopene) compared to other populations and remained stable over time. It was also found that an increase in maternal BMI depletes breastmilk carotenoid concentrations. Our results indicate that the maternal dietary intake of carotenoids is an important factor that influences breastmilk carotenoid concentrations and it is easily modifiable through nutritional intervention. Research should continue to explore the biological impact of such results and improve the knowledge of the unique composition of human milk. These findings may help to determine the nutritional recommendations for the dietary intake of carotenoids for breastfeeding mothers and infants and may also form the basis for the development of nutritional programs or dietary supplements. Moreover, an analysis of breastmilk carotenoids may be useful as a non-invasive method to monitor maternal carotenoid status, as well as its intake by infants. 

## Figures and Tables

**Table 1:** Study design characteristics.

Study Visit		Assessment			
		Breastmilk	Nutritional	Anthropometric	Psychological
1st (1st month)	Mother	- macronutrients - carotenoids - fatty acid profile	- Food Frequency Questionnaire FFQ-6 ^1^ [34] - dietary supplement use	- pre-pregnancy, 14 Hbd ^2^, 27 Hbd body mass - current body mass	- EPDS ^3^ [35] - PSS-10 ^4^ [36,37]
	Infant	-	- breastfeeding frequency - dietary supplement use	- birth parameters - body mass - body length - head circumference	-
2nd (3rd month)	Mother	- macronutrients - carotenoids - fatty acid profile	- 3-day dietary record - carotenoid intake - dietary supplement use	- current body mass	- EPDS ^3^ [35] - PSS-10 ^4^ [36,37]
	Infant	-	- breastfeeding frequency - dietary supplement use	- body mass - body length - head circumference	-
3rd (6th month)	Mother	- macronutrients - carotenoids - fatty acid profile	- 3-day dietary record - carotenoid intake - dietary supplement use	- current body mass	- EPDS ^3^ [35] - PSS-10 ^4^ [36,37]
	Infant	-	- breastfeeding frequency - dietary supplement use - introduced to other foods or drinks	- body mass - body length - head circumference	- DSR ^5^ Scale [38].

^1^ FFQ-6—Food Frequency Questionnaire; ^2^ Hbd—hebdomas (weeks of gestation); ^3^ EPDS—Edinburgh Postpartum Depression Scale; ^4^ PSS-10—Perceived Stress Scale; ^5^ DSR—Children Development Scale.

**Table 2:** Energy value, fat and carotenoid content in breastmilk in the first, third and sixth month of lactation.

Nutrient (Unit)	Breastmilk Composition Mean Value (95% CI ^1^) Min–Max			*p*-Value ^2^
	First Month (*n* = 53)	Third Month (*n* = 49)	Sixth Month (*n* = 47)	
Energy (kcal/100 mL)	69.5 (67.5–71.6) 57.0–89.7	68.2 (65.3–71.0) 49.3–95.5	68.4 (66.5–70.4) 53.7–90.3	0.777
Total fat (g/100 mL)	3.8 (3.6–4.1) 2.2–6.0	3.8 (3.5–4.1) 1.7–6.9	3.9 (3.7–4.1) 2.2–6.4	0.595
β-carotene (nmol/L)	33.2 (33.0–33.5) 31.9–36.3	33.1 (32.9–33.3) 31.8–35.9	33.3 (33.1–33.6) 32.3–35.4	0.436
Lycopene (nmol/L)	112.2 (106.1–118.3) 77.2–169.0	111.2 (105.0–117.3) 75.4–176.9	110.1 (103.9–116.3) 78.3–176.7	0.457
Lutein + zeaxanthin (nmol/L)	33.0 (26.3–39.7) 2.7–123.2	33.0 (24.1–41.8) 3.2–139.9	37.1 (26.5–47.8) 1.8–169.0	0.640

^1^ CI—coefficient interval; ^2^ Friedman ANOVA test.

**Table 3:** Energy value and selected nutrients and carotenoid intake by participants at the third and sixth month of lactation.

Nutrient (Unit)	Dietary Intake Mean Value ± SD ^1^ or (95% CI ^2^) Min–Max		*p*-Value ^3^
	Third Month (*n* = 49)	Sixth Month (*n* = 47)	
Energy (kcal/day)	2193.7 ± 631.17 1186.5–3914.0	2046.2 ± 502.9 1051.4–3317.3	0.083
Total fat (g/day)	84.3 ± 28.5 37.2–185.6	76.4 ± 26.1 18.9–135.7	0.085
Vitamin E, dietary (mg α-tocopherol Equivalent/day)	12.6 ± 5.9 4.3–32.7	11.4 ± 5.1 4.7–24.5	0.258
Vitamin E, dietary & supplements (mg α-tocopherol Equivalent/day)	21.5 ± 13.9 4.3–57.7	13.1 ± 6.7 4.7–27.5	0.000
Vitamin A, dietary (µg Retinol Equivalent/day)	1289.5 ± 591.4 226.4–2447.3	1030.1 ± 500.0 213.1–2745.2	0.010
Vitamin A, dietary & supplements (µg Retinol Equivalent/day)	1295.0 ± 588.3 226.4–2447.3	1030.1 ± 500.0 213.1–2745.2	0.009
β-carotene (µg/day)	4480.8 (3575.0–5386.7) 319.6–16461.0	3441.9 (5037.5–9474.1) 716.2–9552.9	0.232
Lycopene (µg/day)	7897.3 (5465.2–10329.5) 477.2–30472.7	7255.8 (5037.5–9474.1) 339.2 – 38852.9	0.422
Lutein + zeaxanthin (µg/day)	2945.2 (1910.8–3979.6) 263.7–16678.9	3739.3 (2834.9–4643.7) 128.9–12207.3	0.054

^1^ SD—standard deviation; ^2^ CI—coefficient interval; ^3^ Wilcoxon matched pairs test.

**Table 4:** Correlations between breastmilk carotenoids and maternal characteristics and dietary intake.

Variables	Breastmilk β-carotene *r *Coefficient (*p*-Value)		Breastmilk Lycopene *r *Coefficient (*p*-Value)		Breastmilk Lutein + Zeaxanthin *r *Coefficient (*p*-Value)	
	Third Month (*n* = 49)	Sixth Month (*n* = 47)	Third Month (*n* = 49)	Sixth Month (*n* = 47)	Third Month (*n* = 49)	Sixth Month (*n* = 47)
Maternal characteristic						
Maternal age (years)^ 1^	0.015	−0.197	−0.136	0.113	−0.048	−0.009
Maternal education ^2^	0.022	−0.091	0.026	0.046	−0.029	−0.020
Mode of delivery ^2^	−0.009	−0.001	−0.094	−0.050	−0.175	−0.115
Number of children ^2^	0.079	0.061	−0.003	0.105	0.057	0.037
BMI at third or sixth month (kg/m^2^)^ 1^	−0.248 *	−0.337 *	−0.286 *	−0.119	−0.355*	−0.205
Maternal dietary intake						
Carotenoid intake (µg) ^1,3^	0.442 ***	0.532 ***	0.374 **	0.338 *	0.711 ***	0.726 ***
Energy intake (kcal/day)^ 1^	0.041	0.182	−0.097	−0.005	−0.078	0.169
Fat intake (g/day)^ 1^	0.049	0.302 *	−0.197	−0.155	−0.105	0.167
Vitamin E intake (mg/day) ^1^	0.270	0.062	0.082	0.013	0.123	−0.069
Vitamin A intake (µg/day)^ 1^	0.008	0.136	0.175	0.241	−0.027	−0.001
Breastmilk composition						
Breastmilk fat (g/100 mL)^ 1^	0.235	0.167	0.274	0.064	0.399 *	0.237

^1^ Spearman rank correlation coefficient; ^2 ^*tau* Kendall coefficient; ^3^ intake of respective carotenoid; * *p* ≤ 0.05; ** *p* ≤ 0.01; *** *p* ≤ 0.001.

**Table 5:** Univariate and multivariate linear regression models between breastmilk carotenoids and dietary intake and third and sixth months of lactation.

Model	Dietary Intake	Breastmilk Carotenoids at Third Month of Lactation			Breastmilk Carotenoids at Sixth Month of Lactation		
		β-carotene (*n* = 49)	Lycopene (*n* = 49)	Lutein + Zeaxanthin (*n* = 49)	β-carotene (*n* = 47)	Lycopene (*n* = 47)	Lutein + Zeaxanthin (*n* = 47)
1	β (95% CI) *p*-value	0.342 (0.066–0.618) 0.016	0.364 (0.087–0.640) 0.011	0.711 (0.504–0.917) 0.000	0.397 (0.121–0.672) 0.016	0.364 (0.084–0.643) 0.012	0.779 (0.591–0.967) 0.000
	Model parameters	*R*^2^ = 0.10	*R*^2 ^= 0.11	*R*^2 ^= 0.49	*R*^2 ^= 0.14	*R*^2 ^= 0.11	*R*^2 ^= 0.60
2	β (95% CI) *p*-value	0.325 (0.054–0.596) 0.020	0.369 (0.088–0.650) 0.011	0.680 (0.468–0.891) 0.000	0.391 (0.116–0.665) 0.006	0.379 (0.092–0.665) 0.011	0.785 (0.593–0.977) 0.000
	Model parameters	*R*^2^ = 0.14 0.013	*R*^2^ = 0.10 0.039	*R*^2^ = 0.50 0.000	*R*^2^ = 0.07 0.037	*R*^2^ = 0.10 0.037	*R*^2^ = 0.60 0.000
3	β (95% CI) *p*-value	0.325 (0.054–0.596) 0.020	0.369 (0.088–0.650) 0.011	0.680 (0.468–0.891) 0.000	0.391 (0.116–0.665) 0.006	0.379 (0.092–0.665) 0.011	0.785 (0.593–0.977) 0.000
	Model parameters	*R*^2^ = 0.06 0.238	*R*^2^ = 0.11 0.126	*R*^2^ = 0.62 0.000	*R*^2^ = 0.29 0.005	*R*^2^ = 0.07 0.191	*R*^2^ = 0.62 0.000
4	β (95% CI) *p*-value	0.407 (0.094–0.721) 0.012	0.415 (0.104–0.726) 0.010	0.730 (0.516–0.943) 0.000	0.428 (0.180–0.676) 0.001	0.401 (0.089–0.713) 0.013	0.644 (0.448–0.840) 0.000
	Model parameters	*R*^2^ = 0.04 0.337	*R*^2^ = 0.06 0.262	*R*^2^ = 0.51 0.000	*R*^2^ = 0.35 0.003	*R*^2^ = 0.03 0.351	*R*^2^ = 0.68 0.000

Model 1: univariate analysis; Model 2: multivariate analysis adjusted for season; Model 3: multivariate analysis adjusted for maternal age, BMI, education and mode of delivery; Model 4: multivariate analysis adjusted for maternal age, BMI, education and mode of delivery, fat and vitamin A and E intake.

## Supplementary Materials

The following are available online at http://www.mdpi.com/2072-6643/11/1/193/s1, Table S1: Characteristics of the study group, Figure S1: Standard curves for all carotenoids that were used in the presented manuscript.
