# Supplementary material for: Carotenoid Content in Breastmilk in the 3rd and 6th Month of Lactation and Its Associations with Maternal Dietary Intake and Anthropometric Characteristics
Source: Nutrients. 2019 Jan 18;11(1):193. doi: 10.3390/nu11010193 (PMC6356523; doi:10.3390/nu11010193)
Supplement: Supplementary file 1 [file nutrients-11-00193-s001.pdf]

nutrients

MDPI

Carotenoid Content in Breastmilk in the 3rd and 6th Month of Lactation and its Associations with Maternal Dietary Intake and Anthropometric Characteristics

Monika A. Zielinska , Jadwiga Hamulka, Aleksandra Wesolowska

Table S1. Characteristics of the study group.

Variables	1st study visit n=53 (100%) Mean ± SD[1] min – max n (%)	3rd study visit n=47 (89%) Mean ± SD[1] min – max n (%)
**Maternal age (years)**	31.4 ± 3.8<br>23 - 40	31.1 ± 3.6<br>23 – 40
**Maternal education (%)**		
- secondary school or university student	3 (6)	2 (4)
- university, graduated	50 (94)	45 (96)
**Parity (%)**		
- primiparous	25 (47)	22 (47)
- multiparous	28 (53)	25 (53)
**Average income per capita (%)**		
- < 1000 PLN	4 (8)	4 (8)
- 1000 – 1500 PLN	12 (21)	10 (21)
- >1500 PLN	37 (71)	33 (71)
**Civil status (%)**		
- non-formal relationship	9 (17)	7 (15)
- marriage	44 (83)	40 (85)
**Maternal prepregnancy BMI (kg/m²)**	22.3 ± 3.8<br>18.4 – 38.6	22.4 ± 3.6<br>18 – 39
**Maternal prepregnancy nutritional status (%)**		
- underweight	1 (2)	1 (2)
- normal weight	46 (87)	41 (87)
- overweight or obesity	6 (11)	5 (11)
**Maternal BMI at 1st month of lactation (kg/m²)**	23.8 ± 3.6<br>18.4 – 39.7	23.8 ± 3.8<br>18.4 – 39.7
**Maternal nutritional status at 1st month of lactation (%)**		
- underweight	1 (2)	1 (2)
- normal weight	40 (78)	37 (79)
- overweight or obesity	10 (20)	9 (19)
**Maternal BMI at 3rd month of lactation (kg/m²)**	23.2 ± 3.8<br>18.1 – 40.8	23.3 ± 3.8<br>18.1 – 40.8
**Maternal nutritional status at 3rd month of lactation (%)**		

- underweight	1 (2)	1 (2)
- normal weight	42 (86)	40 (85)
- overweight or obesity	6 (12)	6 (13)
**Maternal BMI at 6th month of lactation (kg/m^2)**	22.8 ± 4.0<br>19.0 – 42.6	22.8 ± 4.0<br>19.0 – 42.6
**Maternal nutritional status at 6th month of lactation (%)**		
- underweight	-	-
- normal weight	40 (85)	40 (85)
- overweight or obesity	7 (15)	7 (15)
**Mode of delivery (%)**		
- vaginal	27 (51)	25 (53)
- vaginal with vacuum or forceps use	2 (4)	1 (2)
- planned cesarean section	13 (25)	11 (23)
- emergency cesarean section	11 (21)	10 (21)
**Infant gender (%)**		
- female	28 (53)	25 (53)
- male	25 (47)	22 (47)
**Gestational age (weeks)**	39.3 ± 1.2<br>37 - 42	39.3 ± 1.3<br>37 - 42
**Birthweight (g)**	3391.4 ± 357.5<br>2730.0 – 4240.0	3383.0 ± 370.4<br>2730.0 – 4240.0
**Birth length (cm)**	54.0 ± 2.5<br>47 – 59	54.0 ± 2.6<br>47 – 59

[1] SD – standard deviation.

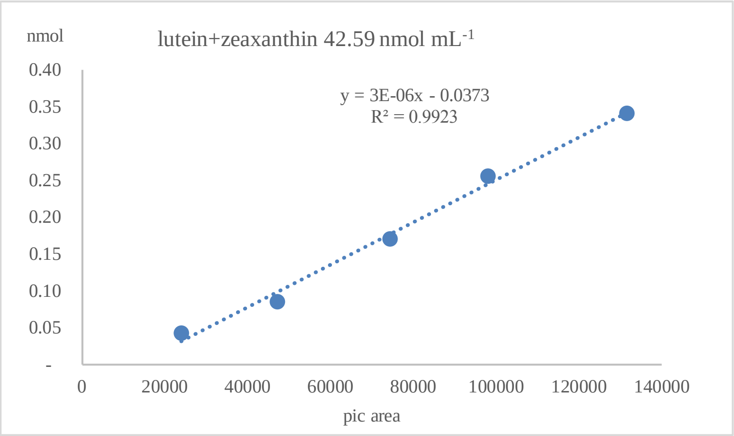

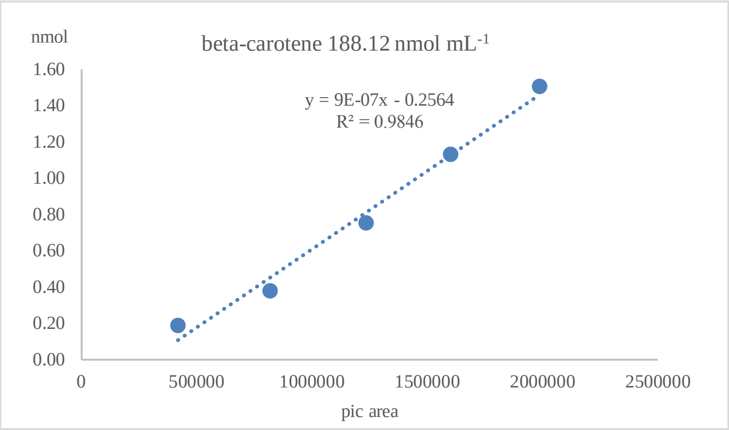

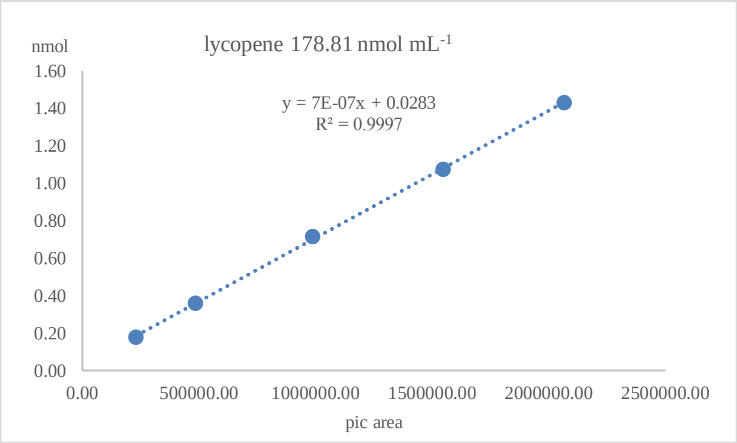

Figure S1: The standard curves for all carotenoids used in presented manuscript.
